# Sex differences in acetylcholine-induced sweating responses due to physical training

**DOI:** 10.1186/1880-6805-33-13

**Published:** 2014-05-29

**Authors:** Yoshimitsu Inoue, Tomoko Ichinose-Kuwahara, Chie Funaki, Hiroyuki Ueda, Yutaka Tochihara, Narihiko Kondo

**Affiliations:** 1 Laboratory for Human Performance Research, Osaka International University, 6-21-57 Tohda-cho, Moriguchi, Osaka 570-8555, Japan; 2 Osaka Shin-ai College, Tsurumi-ku, Osaka 538-0053, Japan; 3 Kyushu University, Fukuoka 815-8540, Japan; 4 Kobe University, Nada-ku, Kobe 657-0053, Japan

**Keywords:** Sweating, Sex, Physical training, Acetylcholine, Iontophoresis

## Abstract

**Purpose:**

The present study examined sex differences in the sweat gland response to acetylcholine (ACh) in physically trained and untrained male and female subjects.

**Methods:**

Sweating responses were induced on the forearm and thigh in resting subjects by ACh iontophoresis using a 10% solution at 2 mA for 5 min at 26°C and 50% relative humidity.

**Results:**

The ACh-induced sweating rate (SR) on the forearm and thigh was greater in physically trained male (*P* < 0.001 for the forearm and thigh, respectively) and female (*P* = 0.08 for the forearm, *P* < 0.001 for the thigh) subjects than in untrained subjects of both sexes. The SR was also significantly greater in physically trained males compared to females at both sites (*P* < 0.001) and in untrained males compared to females on the thigh (*P* < 0.02) only, although the degree of difference was greater in trained subjects than in untrained subjects. These sex differences can be attributed to the difference in sweat output per gland rather than the number of activated sweat glands.

**Conclusion:**

We conclude that physical training enhances the ACh-induced SR in both sexes but that the degree of enhancement is greater in male than in female subjects. The effects of physical training and sex on the SR may be due to changes in peripheral sensitivity to ACh and/or sweat gland size.

## Introduction

One of the effector responses to imposition of internal (exercise) or external heat stress is sweating. Physical training improves sweating responses [1-5], resulting in an increase of sweating rate at a given core temperature. Measurement of the frequency of sweat expulsion [6] and methylcholine injection tests [2] suggest that physical training improves the sensitivity of peripheral mechanisms. However, these results have been obtained primarily from male subjects. Thus, sex differences in sweating enhancement induced by long-term physical training are not fully understood.

The secretion of reproductive hormones is enhanced at puberty, leading to sex differences in physical characteristics and functions. It has been reported that testosterone enhances while estradiol inhibits the sweating response [7]. Testosterone is increased by physical training [8], but the degree of increase in testosterone caused by physical training is smaller in female subjects than in male subjects [9]. These results suggest that the effects of physical training on sweating should be more marked in male than in female subjects.

By examining the sweating rate (SR) and sweat output per gland (SGO) during graded exercise among physically trained and untrained male and female subjects, Ichinose-Kuwahara *et al*. [10] observed that the degree of improvement in sweating response was smaller in female than in male subjects, and that this sex difference was more pronounced with increased exercise intensity. These findings have been challenged by Schwiening *et al*. [11], who suggested that differences in absolute exercise intensity and maximal oxygen uptake (VO_2max_) among groups produced the group differences in SR. However, their challenge remains controversial [12]. The sweat response during exercise is difficult to analyze because it is influenced by various factors, including the degree of thermogenesis during relatively intense exercise, elevation in body temperature during absolute intensity exercise, and physiological functioning of the sweat gland itself. To confirm our previous findings with respect to sex differences in the effect of physical training on sweat responses [10], more accurate measurements of sweating responses using a pharmacological stimulus [2,13,14], which excludes factors associated with exercise, might offer significant information.

In this study, we compared the sweating response to acetylcholine (ACh) on the forearm and thigh in physically trained and untrained male and female subjects to examine the effects of physical training on sex differences related to peripheral sweat glandular sensitivity.

## Methods

### Subjects

In total, 68 volunteers, including 21 physically trained female (TF), 21 untrained female (UF), 14 physically trained male (TM), and 12 untrained male (UM) subjects, participated in this study. The physiological characteristics of the subjects are shown in Table 1. Except for gymnastics lessons, the untrained subjects had not performed regular physical activity for the previous 3 years, while the trained subjects had participated in endurance sports throughout the year for more than 6 years. The trained male subjects belonged to a track club (long- or middle-distance running team) and the trained female subjects belonged to a track (long- or middle-distance running team) or softball club at university. The male and female runners had trained 2 to 3 h/day on 5 days/wk and the female softball players had trained 4 to 5 h/day on 6 days/wk. No female subjects had taken oral contraceptives, which contain female reproductive hormones, and all had self-reported regular menstrual cycles of about 28 days. The purpose and procedures of the study were explained to the subjects prior to the study, and their informed consent was obtained. This study was carried out in accordance with the Declaration of Helsinki and was approved by the ethics committee for human investigation of Osaka International University (Osaka, Japan).

**Table 1:** Physical characteristics of each exercise group

	**Age,**	**Height,**	**Mass,**	**AD/mass,**	**MSF,**	**VO**_**2**_**max,**
	**years**	**cm**	**kg**	**cm**^**2**^**/kg**	**mm**	**ml/kg/minute**
**TF**	21.2 ± 0.3	158.7 ± 1.5^a^	53.1 ± 1.4^a^	282.1 ± 3.1	13.4 ± 0.5^ab^	50.4 ± 1.4^ab^
**UF**	21.6 ± 0.3	162.1 ± 1.1^a^	53.5 ± 1.3^a^	284.7 ± 3.4	18.0 ± 0.8^a^	38.3 ± 1.0
**TM**	20.8 ± 0.4	169.3 ± 1.5	60.2 ± 2.9	275.8 ± 4.8	8.7 ± 0.9	56.4 ± 1.6^b^
**UM**	21.7 ± 0.3	173.8 ± 1.2	61.0 ± 2.0	277.5 ± 4.6	9.5 ± 1.1	37.6 ± 1.8

The values are means ± standard error of the mean for 21 young trained female (TF), 21 young untrained female (UF), 14 young trained male (TM), and 12 young untrained male (UM) subjects. ^a^Significant difference between the sexes for each training status (*P* <0.05). ^b^Significant difference between the untrained and trained subjects of each sex. AD, body surface area; MSF, mean skinfold thickness; VO_2max_, maximal oxygen uptake.

### Protocol

The subjects wore a minimal amount of clothing consisting of a sports bra, short pants, and shorts for female subjects and short pants and briefs for male subjects. After measuring his or her height, body mass, and skinfold thickness, the subject entered an environmental chamber (SRH-30VEVI2; Nagano Science, Osaka, Japan), which was maintained at 26°C and 50% relative humidity. The subject stayed in a sitting position on a chair while the measurement devices were applied. After 40 minutes of stabilization, the ACh iontophoresis test was conducted in the resting subject. The VO_2max_ for each subject was estimated in a submaximal step-load cycle exercise test, performed after the ACh iontophoresis test. All of the female subjects performed the ACh iontophoresis test during the early or mid-follicular phase, defined as 4 to 10 days after the onset of menstruation, to control the effect of the menstrual cycle on heat-loss responses [15,16]. The entire study was conducted between late August and late October, so that the subjects were naturally acclimatized to heat.

### Measurements

The body surface area of each subject was calculated based on his or her height and mass according to the method of Fujimoto and Watanabe [17]; in addition, the percent body fat was assessed as the mean value of the skinfold thickness measured with skinfold calipers over the chest, flank, back, triceps, front of the forearm and thigh, and back of the lower leg.

To estimate the VO_2max_, each subject performed submaximal exercise at four different intensities on a cycle ergometer pedaling at a constant frequency of 50 rpm for 5 minutes without rest between exercise periods. The submaximal exercise was selected to secure the safety of untrained subjects. The VO_2_ (AE300S; Minato Medical Science Co., Ltd, Osaka, Japan) and heart rate (HR: DS-7500; Fukuda Denshi Co., Ltd, Tokyo, Japan) were measured during the final minute of each exercise period, after which the VO_2max_ was estimated for each subject by extrapolating the relationship between VO_2_ and HR to the estimated maximal HR.

Iontophoresis (Iontophoresis CI-5.0; Sukinosu Co., Ltd., Nagoya, Japan) was performed on the skin surface of the forearm and thigh. Two capsules (2.613 cm^2^) for transporting ACh to the skin and for measuring the ACh-induced sweating response were prepared: a 10% ACh solution absorbed into the sponge in the first capsule and ACh transported iontophoretically at 2 mA into the skin of the forearm or thigh for 5 minutes. Immediately after cessation of the current, the capsule was detached, the skin area under the capsule was wiped dry, and the capsule was replaced by a second capsule. Sweating was measured continuously for 7 minutes immediately after the iontophoresis current was stopped using the second capsule. The exchange procedures for the capsules took less than 20 seconds. Sweat production was determined using the capacitance hygrometer-ventilated capsule method [10,18]. In brief, nitrogen gas was passed through the second capsule at a constant flow rate of 300 ml/minute, and the change in relative humidity of the effluent gas was detected by means of a hygrometer (HMP 133Y; Vaisala, Helsinki, Finland). The hygrometer output signals for sweating were recorded at a sampling rate of 50 Hz, and data were captured and stored via a data logger (model MP 100WS; Biopac, Goleta, CA, USA). The mean SR (in mg/cm^2^/minute) was calculated using the data for the last 5 minutes of measurement. Next, the activated sweat gland (ASG) density was determined using the starch-iodide technique [19], and the SGO was calculated by dividing the SR by the ASG.

The oral (T_or_) and skin (T_sl_; at the forearm or thigh) temperatures were measured immediately before iontophoresis using thermistor probes (SZL64; Takara Thermistor Instruments, Yokohama, Japan). The T_or_ and T_sl_ were taken every 30 seconds with a computer-controlled data logger (model K722; Takara Thermistor Instruments). The T_or_ was determined as the temperature at which no change was observed in consecutive T_or_ measurements after the probe had been held sublingually in a closed mouth for at least 5 minutes. The measurement of T_sl_ was made by affixing the probe to the skin with surgical tape (Micropore TM, Surgical tape 1530–1).

### Data analysis and statistics

The primary effects of training status and sex differences were analyzed across conditions using one-way repeated measures analysis of variance. Tukey post-hoc test was used to identify differences among group means. The relationships between VO_2max_ and ACh-induced SR, ASG, and SGO were analyzed by calculation Pearson product–moment correlation coefficients. All statistical analyses were performed using SPSS statistical software (version 11.5; SPSS, Chicago, IL, USA). All data are reported as means ± standard error of the mean (SEM). Statistical significance was set at *P* <0.05.

## Results

### Effects of physical training

Table 1 shows the physical characteristics of the subject groups. No group differences were observed in age, height, mass, and ratio of skin surface area to mass (AD/mass) between the trained and untrained groups for each sex. Mean skinfold thickness (MSF) were significantly lower (*P* <0.001) in the TF group than in the UF group, and VO_2max_ was significantly higher (*P* <0.001) in the TF and TM groups than in the UF and UM groups. The baseline T_or_ and T_sl_ on the forearm or thigh was also not different between the trained and untrained groups for each sex (Table 2).

**Table 2:** **Baseline oral (T**_**or**_**) and skin (T**_**sl**_**) temperatures on the forearm and thigh**

	**T**_**or, **_**°C**	**T**_**sl **_**on the forearm, °C**	**T**_**sl **_**on the thigh, °C**
**TF**	36.56 ± 0.06	32.85 ± 0.15^a^	31.97 ± 0.27
**UF**	36.71 ± 0.07	32.83 ± 0.13	32.20 ± 0.13
**TM**	36.76 ± 0.06	33.50 ± 0.19	32.72 ± 0.20
**UM**	36.70 ± 0.08	33.34 ± 0.20	32.89 ± 0.22

The values are means ± standard error of the mean for 21 young trained female (TF), 21 young untrained female (UF), 14 young trained male (TM), and 12 young untrained male (UM) subjects. ^a^Significant difference between the sexes for each training status (*P* <0.05).

**Figure 1:**
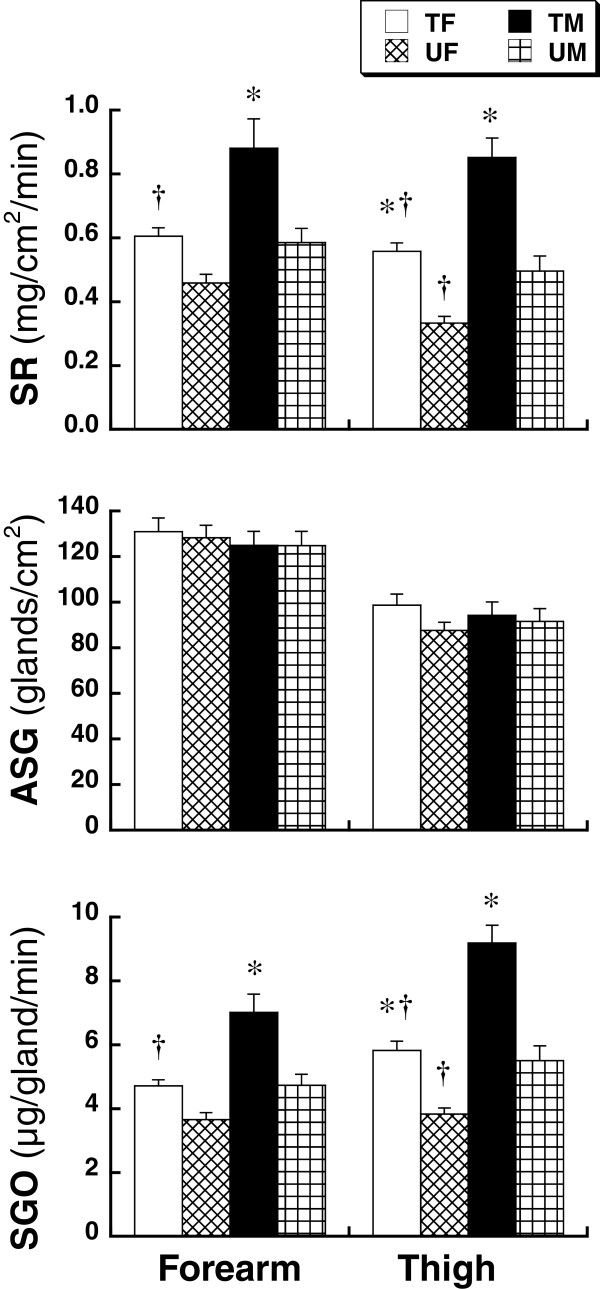
**Sweating rate (SR), number of active sweat glands (ASG), and sweat gland output (SGO) on the forearm and thigh induced by acetylcholine (ACh) applied iontophoretically in physically trained female (TF) or male (TM) subjects and untrained female (UF) or male (UM) subjects.** The values are means ± standard error of the mean (SEM). *Significant difference between the untrained and trained subjects. ^†^Significant difference between the sexes for each training status (*P* <0.05).

The ACh-induced SR and SGO on the thigh of the female subjects were significantly greater (*P* <0.001) in the TF group (SR and SGO: 0.559 ± 0.026 mg/cm^2^/minute and 5.822 ± 0.293 μg/gland/minute) than in the UF group (SR and SGO: 0.333 ± 0.021 mg/cm^2^/minute and 3.828 ± 0.195 μg/gland/minute), but there was a tendency, albeit not significant, towards greater SR (*P* = 0.08) and SGO (*P* = 0.06) on the forearm in the TF group (SR and SGO: 0.605 ± 0.026 mg/cm^2^/minute and 4.716 ± 0.191 μg/gland/minute) than in the UF group (SR and SGO: 0.459 ± 0.027 mg/cm^2^/minute and 3.658 ± 0.223 μg/gland/minute). In contrast, the SR and SGO on the forearm and thigh of the male subjects were significantly greater (*P* <0.001) in the trained group (forearm SR and SGO: 0.881 ± 0.092 mg/cm^2^/minute and 7.015 ± 0.576 μg/gland/minute; thigh SR and SGO: 0.851 ± 0.061 mg/cm^2^/minute and 9.192 ± 0.555 μg/gland/minute) than in the untrained group (forearm SR and SGO: 0.585 ± 0.044 mg/cm^2^/minute and 4.734 ± 0.345 μg/gland/minute; thigh SR and SGO: 0.496 ± 0.047 mg/cm^2^/minute and 5.507 ± 0.460 μg/gland/minute) (Figure 1). Physical training status did not affect the ASG on the forearm and thigh in either sex (forearm and thigh ASG for TF, UF, TM and UM group: 130.9 ± 6.0, 128.3 ± 5.5, 125.0 ± 6.2, and 124.9 ± 6.2 glands/cm^2^, 98.7 ± 4.9, 87.6 ± 3.6, 94.3 ± 5.8, and 91.6 ± 5.6 glands/cm^2^, respectively).

### Effects of sex

There were no sex differences in age or AD/mass due to physical training status (Table 1). The TF and UF groups had significantly lower height (*P* <0.001) and mass (*P* <0.05) and higher MSF values (*P* <0.001) than the TM and UM groups. VO_2max_ was significantly lower in the TF group than the TM group (*P* <0.02), but no such difference was noted between the UF and UM groups (Table 1).

No sex differences were noted in baseline T_or_ and T_sl_ on the thigh due to physical training status (Table 2). The T_sl_ on the forearm was significantly lower (*P* <0.04) in the TF subjects compared to the TM subjects, but no such difference was noted between the UF and UM groups.

In the trained subjects, the SR and SGO on the forearm and thigh were significantly lower (*P* <0.001) in the TF group *versus* the TM group. In the untrained subjects, the SR and SGO were significantly lower (*P* <0.02) in the UF group than the UM group on the thigh, but not on the forearm. There were no sex differences in ASG on the forearm and thigh due to physical training status (Figure 1).

### Relationships between VO_2max_ and ACh-induced sweating

**Figure 2:**
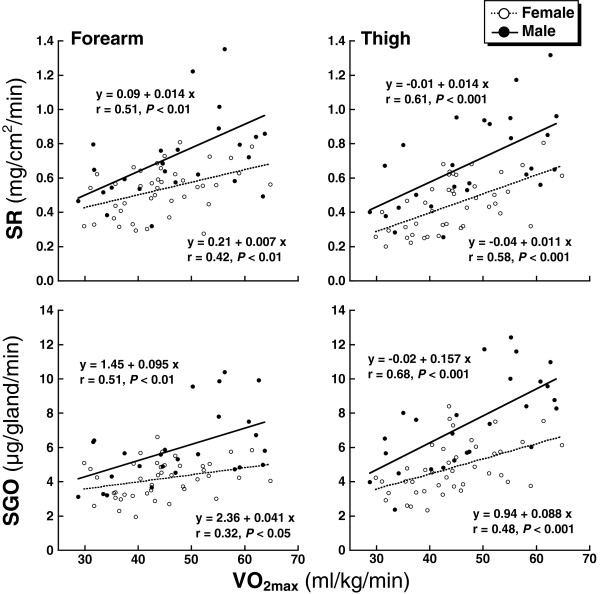
**Correlation between the maximal oxygen uptake (VO**_**2max**_**) and sweating rate (SR) or sweat gland output (SGO) on the forearm and thigh caused by acetylcholine (ACh) iontophoresis in the female (open circles) and male subjects (closed circles).**

Figure 2 depicts the relationships between VO_2max_ and SR or SGO on the forearm and thigh in female and male subjects. The SR and SGO on the forearm and thigh were significantly correlated with the VO_2max_ for each sex (forearm SR and SGO for female: *r* = 0.42, *P* <0.01 and *r* = 0.32, *P* <0.05: forearm SR and SGO for male: *r* = 0.51, *P* <0.01 and *r* = 0.51, *P* <0.01; thigh SR and SGO for female: *r* = 0.58, *P* <0.001 and *r* = 0.48, *P* <0.001; forearm SR and SGO for male subjects: *r* = 0.61, *P* <0.001 and *r* = 0.68, *P* <0.001). The slopes of regression lines were markedly smaller in the female versus male subjects (forearm SR: 0.007 versus 0.014 (mg/cm^2^/minute)/(ml/kg/minute), thigh SR: 0.011 versus 0.014 (mg/cm^2^/minute)/(ml/kg/minute), forearm SGO: 0.041 versus 0.095 (μg/gland/minute)/(ml/kg/minute), thigh SGO: 0.088 versus 0.157 (μg/gland/minute)/(ml/kg/minute)). There was no significant correlation between VO_2max_ and ASG in either sex.

## Discussion

The present study determined that ACh-induced sweating responses were lower in female versus male subjects, and that physical endurance training enhanced the sweating response of both sexes. The degree of sweating response enhancement with endurance training was also smaller in female than in male subjects. These findings suggest that sex differences in peripheral sudomotor sensitivity to ACh were increased by endurance training.

Irrespective of sex, endurance training increased the ACh-induced SR at the tested forearm and thigh. Although ASG was not markedly changed by endurance training status, the SGO and SR were increased in both sexes. These results indicate that endurance training enhances the SR and that this enhanced response can be attributed to SGO improvement and not to changes in the number of ASG. Sato and Sato [13] reported that the sweat glands of subjects judged to be poor sweaters were smaller and had lower secretory activity and cholinergic sensitivity compared with glands from physically fit subjects. Based on this result, it has been suggested that endurance training improves the size or cholinergic sensitivity of sweat glands in female and male subjects, and that there are sex differences in the improvement in size or cholinergic sensitivity of sweat glands.

In the present study, there were remarkable sex differences in SR and SGO due to physical training status, but there was no sex difference in ASG number. These results suggest that the lower SR in the female subjects was not due to a smaller number of ASG, but to a lower SGO. In this regard, the sex differences in SR and SGO observed in the untrained subjects were more pronounced in the trained subjects because the degree of enhancement in SR and SGO with endurance training was smaller in female subjects than in male subjects. For example, the sex difference in SGO on the forearm increased from 1.08 μg/gland/minute in the untrained subjects to 2.30 μg/gland/minute in the trained subjects. Similarly, the sex difference in SGO on the thigh increased from 1.68 μg/gland/minute to 3.37 μg/gland/minute. We also found that VO_2max_, which affects sweating capacity, was significantly lower in the TF than the TM (Table 1), although it was not different between the UF and UM. Therefore, sex differences in VO_2max_ might be partially responsible for the greater sex differences in SR and SGO. In contrast, SR and SGO were significantly correlated with VO_2max_ in both sexes, and the regression analysis lines were markedly lower with smaller slopes in the female subjects than the male subjects. These data strongly suggest that peripheral sweat glandular function is enhanced by physical training in female subjects, although the degree of enhancement is lower in female than in male subjects. This finding is in agreement with previous data showing that the degree of increased exercise-induced SGO with physical training was smaller in female than in male subjects [10]. The present findings strongly contradict the conclusion of Schwiening *et al*. [11]; namely, that the differences in absolute exercise intensity and VO_2max_ result in sex differences in SR during exercise.

Kawahata [7] reported that testosterone enhanced, while estradiol inhibited, the sweating response. Subsequent studies showed that physical training increased testosterone in both sexes [8], but the increase was considerably smaller in female than in male subjects [9]. In the light of these results, our present finding that SR and SGO were similarly enhanced by endurance training to a greater degree in male than in female subjects suggests the possible involvement of testosterone in this enhancement, although testosterone levels were not measured in this study.

Sweating in response to pharmacological stimulation is affected by the local skin temperature [20]. However, since there were no differences in the baseline T_sl_ on the forearm and thigh between the trained and untrained groups of either sex, local skin temperature does not appear to contribute to the higher ACh-induced SR in the trained group versus the untrained group. The sex differences in baseline T_sl_ on the forearm for training status were similar to the sex differences in SR. Therefore, although we cannot exclude the possibility that the sex difference in ACh-induced SR on the forearm observed in the present study was influenced by differences in T_sl_ regardless of physical status, the sex differences that we observed might reflect general differences in sweating since the differences were less than 0.7°C.

In summary, although the sweating responses to ACh were lower in female but not male subjects, endurance training improved these responses regardless of sex. Sex differences do exist in the degree of ACh-induced sweating responses with endurance training since the degree of enhancement was smaller in the female versus male subjects. Therefore, we conclude that sex differences in ACh-induced sweating responses are increased by endurance training. In terms of biological significance, our present findings of lower sweat gland function in female subjects, together with previous findings of their lower body fluid content, allow us to speculate that they might control body fluid loss by reducing excessive sweat in exercise and/or a hot environment. More detailed examination of sex differences in effective and ineffective (dripping) sweating rates during exercise should help clarify this point.

## Abbreviations

ACh: acetylcholine; AD: body surface area; ASG: activated sweat gland; HR: heart rate; MSF: mean skinfold thickness; SR: sweating rate; SGO: sweat output per gland; TF: trained female subjects; TM: physically trained male subjects; T_or_: oral temperature; T_sl_: skin temperature; UF: untrained female subjects; UM: untrained male subjects; VO_2max_: maximal oxygen uptake.

## Competing interests

The authors declare that they have no competing interests.

## Authors’ contributions

YI contributed to the conception and design of study, and wrote the manuscript. TI, CF and HU contributed to the data collection and data analysis. NK and YT edited the manuscript and did the data interpretation. All authors read and approved the final manuscript.
